# Chimeric antigen receptor T‑cell therapy—a hematological success story

**DOI:** 10.1007/s12254-018-0409-x

**Published:** 2018-06-06

**Authors:** Philipp Wohlfarth, Nina Worel, Georg Hopfinger

**Affiliations:** 1 Division of Blood and Marrow Transplantation, Department of Medicine I, Medical University of Vienna, Waehringer Guertel 18–20, 1090 Vienna, Austria; 2 Department of Blood Group Serology and Transfusion Medicine, Medical University of Vienna, Vienna, Austria

**Keywords:** Chimeric antigen receptor T cells, CAR T cells, Adoptive T‑cell transfer, Immunotherapy

## Abstract

Chimeric antigen receptor (CAR) T cells are genetically engineered autologous cells that express an activating receptor targeted towards one or more tumoral antigens. After *ex vivo* production and re-infusion, they are able to proliferate in the host and to recognize and kill tumor cells. Together with checkpoint inhibition, this new therapy is already being celebrated as a major medical breakthrough in recent years, due to the substantial benefit observed in clinical trials with patients with chemotherapy-refractory B‑cell malignancies. These results have led to the recent approval of two CAR T‑cell products by the Food and Drug Administration (FDA) in the United States. The list of targetable antigens and possible indications is continuously being expanded, as are the modifications to the CAR structure and the final cell products currently under investigation. In some patients, CAR T‑cell therapy may lead to substantial toxicity including the cytokine release syndrome (CRS). In summary, CAR T‑cell therapy has already provided clinical benefit to patients with B‑cell malignancies unresponsive to conventional treatment. Yet, the therapy is still in an early stage of development, and the many opportunities for improvement in its various aspects as well as its future role in relation to conventional therapy will set the pace in the field of hematology for the next years or even decades.

## Introduction

Advances in cancer immunotherapy, the effort to harness the immune system to battle tumors, are considered a major medical breakthrough of the recent years. Recently, we witnessed the first approvals of a ground-breaking new form of cell-based immunotherapy, chimeric antigen receptor (CAR) T cells, by the Food and Drug Administration (FDA) in the United States. In Europe, CAR T‑cell products from several pharmaceutical companies have been filed and granted access to the European Medicines Agency (EMA) Priority Medicine (PRIME) scheme to facilitate their approval based on promising results from phase I/II trials. In this article, we give an overview about the principles of CAR T‑cell therapy, review the currently available clinical data, and provide a short overview of future perspectives.

## CAR T‑cell therapy—principles and specifications

T cells can be genetically engineered *ex vivo* to express a chimeric antigen receptor (CAR) in addition to their natural T‑cell receptor (TCR). When one or more tumor-specific antigens are targeted, T cells harboring the CAR are able to proliferate and kill tumor cells upon antigen recognition. In contrast to the natural T‑cell response, this process is not major histocompatibility complex (MHC) restricted but only dependent on the presence of the targeted surface antigen, thus, eliminating MHC downregulation as a major mechanism of cancer immune evasion [1].

**Fig. 1:**
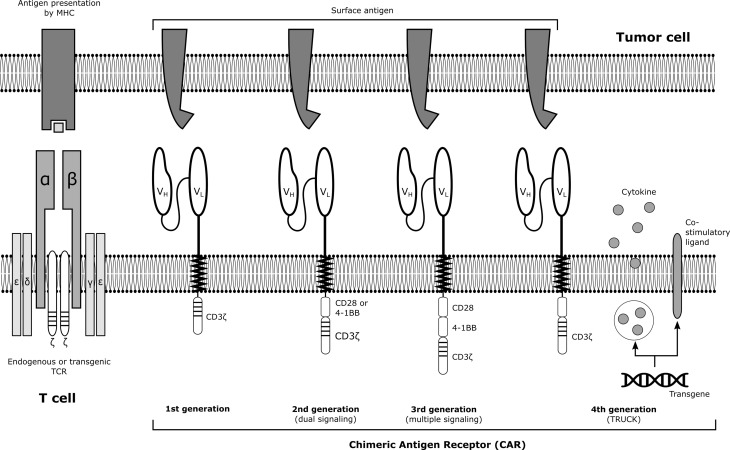
Schematic illustration of the endogenous T‑cell receptor (TCR) and chimeric antigen receptor (CAR) constructs. Malignant cells present tumoral antigens via major histocompatibility (MHC) molecules that are recognized by the endogenous TCR. CAR T‑cells recognize targeted surface antigens (e. g., CD19) via their ligand binding domain derived from a monoclonal antibody (V_H_ and V_L_; variable heavy and light chain). The antigen recognition moiety is linked to a transmembrane domain with a hinge fragment. All CAR constructs contain the CD3ζ signaling domain. Second or later generation CARs further contain one or more co-stimulatory domains (e. g., CD28 or 4‑1BB), enhancing the cytotoxic response of the transfected cell upon antigen recognition. T cells redirected for universal cell killing (TRUCKs) release cytokines or express co-stimulatory ligands upon antigen stimulation with the intent to augment activation and to attract cells of the innate immune system. Adapted from [2]

CARs are fusion proteins that are composed of an extracellular binding domain, a hinge region, a transmembrane domain, and one or more intracellular signaling domains (Fig. 1; [2]). The antigen-recognition moiety is commonly a single-chain variable fragment (scFv) derived from a tumor-antigen reactive monoclonal antibody. Targeting of B‑cell antigen CD19 to treat acute lymphoblastic leukemia (ALL) and non-Hodgkin lymphoma (NHL) has so far produced the most considerable clinical success rates [3], but a variety of other antigens are currently under consideration for targeting of malignant hematologic diseases (Table 1).

**Table 1:** List of selected target antigens with available trial results or currently under investigation

Antigen	Disease	Trial/Product and clinical results
CD19	B-cell malignancies	*KTE-C19/Axicabtagene ciloleucel (NHL)*^*a*^*:* 82% ORR, 54% CR, 42% with ongoing response after median follow-up of 15.4 months (ZUMA-1, *n* = 111) [7]
		*CTL-019/Tisagenlecleucel (ALL <21* *y)*^*b*^: 81% CR, all MRD negative, RFS 80% and 59% at 6 and 12 months, respectively (ELIANA, *n* = 75) [11]
		*CTL-019/Tisagenlecleucel (DLBCL)*^*b*^*:* 53% ORR, 40% CR, RFS 74% at 6 months (JULIET, *n* = 99) [14]
		*Park et al. (ALL ≥18* *y):* 83% CR, median EFS 6 months (*n* = 53) [25]
		*JCAR017 (NHL):* 75% ORR, 56% CR, 37% with CR at 6 months (results only reported for DLBCL cohort, *n* = 69) [15]
CD30	Hodgkin lymphoma, T‑cell lymphoma	*Wang et al. (HL, ALCL):* 0/18 CR, 7/18 PR, 1/18 SD, 10/18 NR [26]
		*Ramos et al. (HL, ALCL):* 3/9 CR, 3/9 SD, 3/9 NR [27]
CD22	B-cell malignancies	*Fry et al. (ALL):* 73% CR; CR also in 5/5 pts. with CD19^−^ or CD19^dim^ B‑ALL (*n* = 21) [28]
CD20	B-cell malignancies	*Zhang et al. (NHL):* 82% ORR, 55% CR (*n* = 11) [29]
BCMA	Multiple myeloma	*Bb2121 (MM):* 89% ORR; 100% ORR in pts. with >150 × 10^6 CAR T cells, 3/15 sCR, 1/15 CR, 7/15 VGPR, 4/15 PR [30]

Either the designated CAR T‑cell product reference (*italic*) or, if not applicable, the name of the first-author of the respective publication is shown

*ALCL* anaplastic large cell lymphoma, *BCMA* B-cell maturation antigen, *CR* complete remission, *DLBCL* diffuse large B‑cell lymphoma, *EFS* event-free survival, *HL* Hodgkin lymphoma, *MRD* minimal residual disease, *NHL* non-Hodgkin lymphoma, *NR* no response, *ORR* overall response rate, *PR* partial remission, *RFS* relapse-free survival, *SD* stable disease, *sCR* stringent complete remission, *VGPR* very good partial remission

^a^Marketed as “Yescarta” in the United States

^b^Marketed as “Kymriah” in the United States

CAR T cells are infused intravenously either at a single dose or as split doses in multiple injections. Importantly, CAR T‑cell infusion should be preceded by a lymphocyte-depleting conditioning regimen (e. g., fludarabine in combination with cyclophosphamide), as this was shown to enhance their efficacy by elevating cytokine levels and possibly by reducing the number of inhibitory regulatory T cells (T_regs_) in the host [4, 5]. Following infusion and *in vivo* expansion, CAR T cells have been shown to be able to persist and remain functionally active for several years in some patients [6].

Autologous unselected peripheral blood mononuclear cells (PBMCs) are most commonly used as the starting material for CAR T‑cell generation. T cells are isolated from the apheresis product and usually transfected with the CAR construct by using replication incompetent gamma-retroviruses or lentiviruses. In the final steps, the CAR T cells are expanded and the product is formulated. As CAR T‑cell generation is a delicate process, it is so far only possible in a handful of GMP-certified facilities worldwide. Still, a recently published landmark trial (ZUMA-1) involving 22 centers demonstrated the feasibility of CAR T‑cell generation as a centralized process with a 99% production success rate and a median time from apheresis to delivery of the product to the administration facility of only 17 days [7]. Currently, several pharmaceutical companies pursue CAR-cell therapies (Table 1).

CAR T‑cell therapy may come with significant side effects, some of which can be fatal. Most prominently, a “cytokine release syndrome” (CRS), characterized by fever, tachycardia and hypotension and associated with excessive cytokine release by the CAR T cells in response to tumor recognition has been described already in the first CAR T‑cell trials [8]. Some mild form of CRS can be observed almost universally (up to 90% of patients), but around 15–40% of the patients will experience grade 3/4 CRS and thus require vasopressors and/or respiratory support. While mild cases of CRS may be managed with supportive care, tocilizumab (interleukin-6 antibody) is the drug of choice for the treatment of severe CRS followed by corticosteroids. However, the latter have been shown to lead to CAR T‑cell ablation at least in some patients and are thus considered only second-line therapy [9].

Neurological toxicities such as confusion, tremors, ataxia, and aphasia are another frequently observed complication with reported 30–40% incidence rates, and may occur alone or as part of the CRS. The complications are usually self-limited, but cases of fatal cerebral edemas have also been described. Their pathophysiology is so far not understood. Other toxicities of CAR T‑cell therapy may include “on target, off tumor recognition”, for example, leading to B‑cell aplasia and hypogammaglobulinemia with CD19 CAR T cells, and cytopenia due to the conditioning regimen [10].

## Clinical data

The breakthrough of CAR T cells is closely linked to the use of the B‑lymphocyte differentiation antigen CD19 as a target in B‑cell malignancies. A pooled analysis from CD19 CAR T‑cell trails including 243 patients reported by the end of the year 2016 estimated a 60% response rate with only around 20% of documented non-responders in a relapsed and/or refractory (r/r) or heavy pretreatment setting. Overall, these data suggest that CD19 CAR T cells might be most effective in patients with ALL, less so for B‑NHL and the least for chronic lymphocytic leukemia (CLL) [2]. Consecutive results from multinational phase II studies led to the recent approval of CAR T‑cell products to treat r/r ALL in children and young adults (ELIANA study) [11] and r/r aggressive B‑NHL (ZUMA-1 study) [7] by the FDA.

The ELIANA study is a single-arm multicenter global phase II study investigating the single-infusion of the CTL019 CAR T‑cell product in r/r ALL patients up to 21 years of age. In February 2018, interim data for 92 enrolled patients were published [11]. At that time, 75 out of 92 included patients had been infused with CTL019. Patients in the trial had received a median of three prior lines of therapy and 61% had already received an HSCT. Despite this heavy pretreatment, 81% of the patients attained at least a complete remission (CR) with incomplete blood recovery after a single infusion. Additionally, all of these responding patients had minimal residual-disease negative marrow after treatment. The estimated relapse-free probability at 12 months in responders was 80%. Eight out of the 61 responders (13%) proceeded to allogeneic hematopoietic stem cell transplantation within 6 months while in remission. Grade 3/4 CRS occurred in 46% of the patients, and one associated death was reported.

The ZUMA-1 study is a phase I/II multicenter study enrolling patients with r/r large B‑cell lymphoma (diffuse large B‑cell lymphoma, primary mediastinal B‑cell lymphoma, or transformed follicular lymphoma). Results for 111 patients treated with KTE-C19 CAR T cells as part of the study were recently reported in the *New England Journal of Medicine* [7]. There was an 82% objective response rate that was consistent across all lymphoma subtypes, with 54% of patients achieving a CR. At a median follow-up of 15 months, 42% had a durable response and 40% continued to have a CR. This resulted in a 52% survival rate at 18 months (median overall survival: not reached), which compares favorably to the expected median overall survival of 6 months in patients with refractory DLBCL (70% of the included patients) [12]. Grade 3/4 CRS occurred in 13% of the patients, and two associated deaths were reported. Mature data in B‑NHL are also available for other CAR T‑cell products (Table 1). As an example, Schuster et al. reported an overall response rate of 64% in a case series of 28 patients with r/r B‑NHL treated with CTL-019 and confirmed the durability of remissions in >80% of the patients after a median follow-up of 29 months [13]. Interim results for 99 patients included in the global extension study (JULIET trial) and infused with CTL-019 for treatment of r/r DLBCL were also recently reported in abstract form at the American Society of Hematology (ASH) Congress (53% overall response rate; 30% CR and 7% PR rate at 6 months, respectively) [14]. Importantly, responses were consistent over all subgroups including double-hit lymphoma. Data for the JCAR017 product from patients participating in the TRANSCEND NHL 001 trial [15] were also presented at the same congress and showed similar results as ZUMA-1 and JULIET.

## Perspective

Although already considered a major hematological success, CAR T‑cell therapies are still at an early stage of development and their various facets offer great opportunities for improvements. As examples, modifications in CAR design allow for the creation of armored CAR T cells (Fig. 1) and products with an incorporated suicide gene that would allow their ablation upon stimulation with a pharmacologic agent in cases of life-threatening toxicity. In general, the possible composition and modifications of CAR domains already now give rise to an unlimited number of possible CAR T‑cell products with specific profiles regarding immunogenicity, expansive capabilities, cytokine secretion, cytotoxicity as well as in-host persistence [16]. Hence, it will be a long way to define the ideal CAR construct for each entity and patient. Not all patients respond to CAR T‑cell therapy and relapses rates are significant also with this approach. The latter have in some cases been linked to loss of tumor antigen [17] or immune escape via the PD1-PD-L1 axis [18]. Efforts to overcome primary or secondary resistance thus include multi or tandem CAR T cells targeting two different tumor antigens (e. g., CD19 and CD22) [19], or the use of checkpoint inhibitors [20] or interleukin-15 [21] to reactivate exhausted CAR T cells. Furthermore, several pharmaceutical agents such as ibrutinib have shown to enhance CAR T‑cell efficacy in preclinical models and their supplemental use is currently being investigated [22]. Other possible modifications include the type and intensity of the conditioning regimen, the formulation of the CAR T‑cell product (e. g., selection of T‑cell subsets), the administered cell dose, or their use in earlier disease stages [16]. Finally, advances in genome editing (e. g., CRISPR/Cas9 or TALEN) may allow for the creation of off-the-shelf universal allogeneic CAR T cells with disrupted endogenous TCR expression, thus, diminishing the risk for graft-versus-host reactions [23, 24].

## Conclusion

Adoptive CAR T‑cell therapy is a promising new approach to treat chemotherapy-refractory hematologic cancers and has already proven capability to induce durable complete remissions in patients with ALL and B‑NHL. As the list of targeted antigens and thereby the possible indications will be extended, continuous modifications in the production and composition of CAR T‑cell products as well as pre- and post-administration treatments will set the pace to keep researchers and clinicians involved in the future. Ultimately, as many patients as possible have to benefit from CAR T cell therapy. For this, the alleviation of possible toxicities as well as treatment accessibility in the light of its (current) logistic and economic burdens have to be addressed.

## Abbreviations

ALL: Acute lymphoblastic leukemia
CAR: Chimeric antigen receptor
CD19: Cluster of differentiation 19
CLL: Chronic lymphocytic leukemia
CR: Complete remission
CRS: Cytokine release syndrome
DLBCL: Diffuse large B‑cell lymphoma
EMA: European Medicines Agency
FDA: Food and Drug Administration
MHC: Major histocompatibility complex
NHL: Non-Hodgkin lymphoma
PBMCs: Peripheral blood mononuclear cells
scFv: Single-chain variable antibody fragment
TCR: T-cell receptor
